# Apparent Biological Motion in First and Third Person Perspective

**DOI:** 10.1177/2041669516669156

**Published:** 2016-09-21

**Authors:** Emmanuele Tidoni, Michele Scandola, Veronica Orvalho, Matteo Candidi

**Affiliations:** University of Rome “Sapienza”, Italy; IRCCS, Rome, Italy; IRCCS, Rome, Italy; University of Verona, Italy; Universidade do Porto, Portugal; University of Rome “Sapienza”, Italy; IRCCS, Rome, Italy

**Keywords:** virtual reality, virtual hand illusion, apparent motion, perspective, motor control

## Abstract

Apparent biological motion is the perception of plausible movements when two alternating images depicting the initial and final phase of an action are presented at specific stimulus onset asynchronies. Here, we show lower subjective apparent biological motion perception when actions are observed from a first relative to a third visual perspective. These findings are discussed within the context of sensorimotor contributions to body ownership.

Apparent biological motion (ABM; Shiffrar & Freyd, 1990) allows investigating how the visual system processes observed body movements (Funk, Shiffrar, & Brugger, 2005; Vannuscorps & Caramazza, 2016). Images taken from a third person perspective (3PP) have been typically used, and recent studies with immersive systems investigated action observation and action monitoring mechanisms in first person perspective (1PP; Padrao, Gonzalez-Franco, Sanchez-Vives, Slater, & Rodriguez-Fornells, 2016; Pavone et al., 2016). To date, the role of 1PP in ABM task has not been assessed.

Thirteen healthy right-handed volunteers wore an Head Mounted Display (Oculus DK1) and observed two avatars (Alvarez et al., 2011; Orvalho, Bastos, Parke, Oliveira, & Alvarez, 2012): from a 1PP and a 3PP (Figure 1(a)). Participants assessed the plausibility of the perceived ABM (through vs. above an obstacle) for the right index and little finger, in two separate blocks, by pressing two buttons with the middle and ring finger of the left hand. The initial and final positions of the fingers were presented for 90 ms and five stimulus onset asynchronies (SOAs) (100, 400, 700, 1,000, and 1,300 ms; Funk et al., 2005) gradually increased the perception of seeing the finger moving along a trajectory above an obstacle (Figure 1(a)). Two finger movements enabled to verify the generalizability of the results and describe any possible role of motor dexterity on visual perception (i.e., index finger movements are more familiar than little finger actions; Plata Bello, Modroño, Marcano, & González-Mora, 2013; Plata Bello, Modroño, Marcano, & González-Mora, 2015). Blocks order and response buttons were almost counterbalanced across subjects. There were 80 trials for each block (8 trials for each SOA-Finger interaction; 40 for 1PP; 40 for 3PP). Participants were allowed to watch the stimuli for as long as needed, and “perceived ABM” was collected (i.e., plausible ABM “I perceived the finger as moving over the obstacle” vs. implausible ABM “I perceived the finger as moving through the obstacle”). No visible movements of subjects’ right fingers were noted during the experiment. After each block participants verbally rated on a 7-point rating scale their agreement with a set of questions (−3 = *completely disagree*, 0 = *neither agree nor disagree*, 3 = *completely agree*; see Figure 1(b)) in order to control illusory sensations over the virtual bodies.

**Figure 1.**
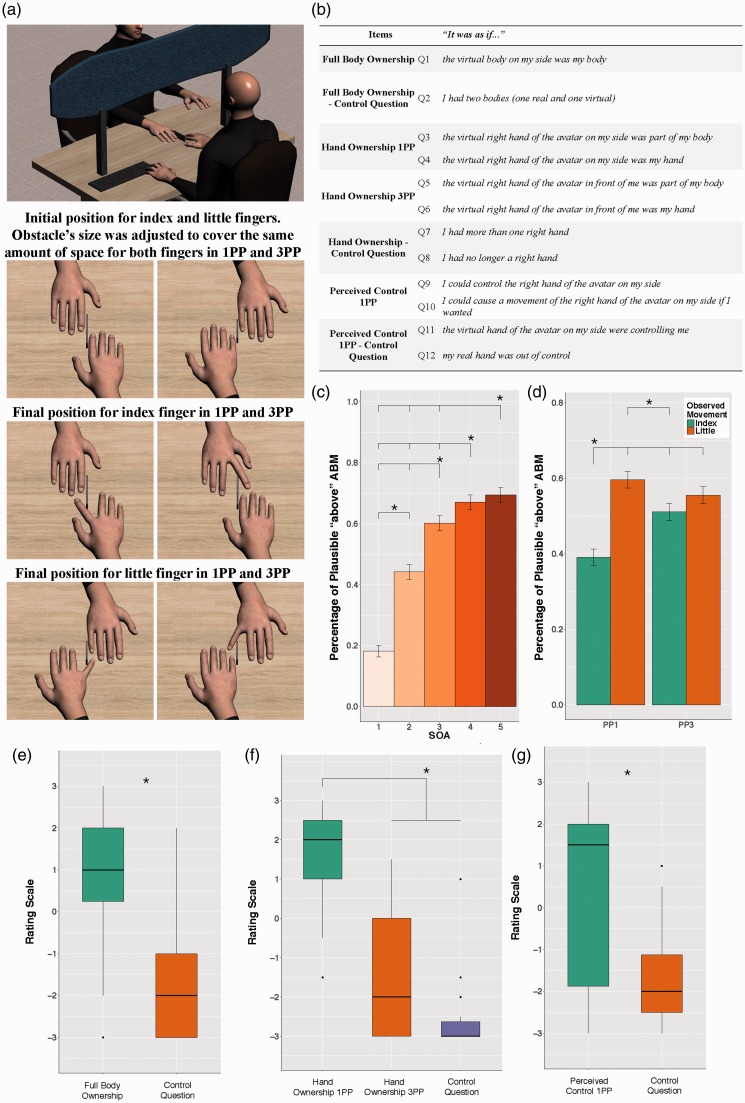
(a) An image showing the virtual environment and the initial and final position for index and little fingers in 1PP and 3PP. Obstacle’s size was adjusted to cover ∼40% of the fingers’ length for both 1PP and 3PP. (b) The items participants answered on a −3 to +3 rating scale. Percentage of plausible “above” ABM as a function of SOA (c) and perspective (d). Error bars indicate standard error mean. (e)–(g) Rating values for each item. The horizontal black bars are the medians, and the boxes are the interquartile ranges (IQRs). Whiskers are within 1.5* IQR, and data beyond the end of the whiskers are plotted as points. All asterisks denote *p* < .05. All *p* are FDR corrected.

Binary ABM answers were analyzed using logistic-GLMER mixed effects regression in “lme4” package (Bates, Maechler, Bolker, & Walker, 2016; R Development Core Team, 2013) with Perspective, SOA, and Finger as fixed effects. Ratings were analyzed using Cumulative Linear Mixed Model (CLMM) in “ordinal” package (Christensen, 2015) with Perspective and Finger as fixed effects. For all multilevel analyses, a by-subjects random intercept was included, and the saturated model (i.e., the model with all the available fixed parameters, factors, and interactions) was simplified by hierarchically dropping effects and interactions with *p* > .1. For the sake of simplicity, we report only the parameters of the final best-fitting model by considering both Akaike information criterion, Bayesian information criterion, and the log likelihood indexes.

Plausible ABM (i.e., above the obstacle) was affected by SOA (*p* < .001, Figure 1(c)), Perspective (*p* < .001, 45.1% for 1PP vs. 57.6% for 3PP). A trend to significance for Finger (*p* = .053) indicated a lower tendency to report plausible ABM (i.e., “above”) for the index (49.5%) relative to the little finger (53.3%). This was accounted for by a significant Perspective × Finger interaction with a lower rate of plausible ABM for Index-1PP relative to all other conditions (all *p* < .001) and Index-3PP relative to Little-1PP (*p* = .011, Figure 1(d)).

Finally, participants experienced full-body-ownership and perceived control (Tieri, Tidoni, Pavone, & Aglioti, 2015a) over the observed movements (Tieri, Tidoni, Pavone, & Aglioti, 2015b) as compared with control questions (all *p* < .001, Figure 1(f) to (h)). Importantly, participants embodied only the virtual hand observed in 1PP compared to the hand in 3PP and to control questions (*p* < .001, Figure 1(g)).

Overall, the present data indicate that ABM perception may be affected by perspective and motor dexterity. That lower ABM was experienced only for the index in 1PP suggests a combined role of motor familiarity (Plata Bello et al., 2013, 2015) and embodiment over ABM perception. Crucially, participants were less prone to report a plausible “above” ABM when the action was observed from a 1PP, and further studies are necessary to disentangle the role of visual perspective from body ownership and the perceived control over the observed movements from a 1PP (Tieri et al., 2015b; Wegner, Sparrow, & Winerman, 2004). Virtual reality represents a useful tool to test the role of bodily re-afferences and sensorimotor brain areas responsible of motion/action perception during perceptual judgments (Orgs et al., 2016; Pavone et al., 2016; Vannuscorps & Caramazza, 2016) when participants are embodied in virtual agents presented from a 1PP.
